# A missense variant in Mitochondrial Amidoxime Reducing Component 1 gene and protection against liver disease

**DOI:** 10.1371/journal.pgen.1008629

**Published:** 2020-04-13

**Authors:** Connor A. Emdin, Mary E. Haas, Amit V. Khera, Krishna Aragam, Mark Chaffin, Derek Klarin, George Hindy, Lan Jiang, Wei-Qi Wei, Qiping Feng, Juha Karjalainen, Aki Havulinna, Tuomo Kiiskinen, Alexander Bick, Diego Ardissino, James G. Wilson, Heribert Schunkert, Ruth McPherson, Hugh Watkins, Roberto Elosua, Matthew J. Bown, Nilesh J. Samani, Usman Baber, Jeanette Erdmann, Namrata Gupta, John Danesh, Danish Saleheen, Kyong-Mi Chang, Marijana Vujkovic, Ben Voight, Scott Damrauer, Julie Lynch, David Kaplan, Marina Serper, Philip Tsao, Million Veteran Program, Josep Mercader, Craig Hanis, Mark Daly, Joshua Denny, Stacey Gabriel, Sekar Kathiresan

**Affiliations:** 1 Center for Genomic Medicine, Massachusetts General Hospital, Boston, Massachusetts, United States of America; 2 Department of Medicine, Harvard Medical School, Boston, Massachusetts, United States of America; 3 Program in Medical and Population Genetics, Broad Institute, Cambridge, Massachusetts, United States of America; 4 Departments of Biomedical Informatics, Vanderbilt University, Vanderbilt, Tennessee, United States of America; 5 Departments of Medicine, Vanderbilt University, Vanderbilt, Tennessee, United States of America; 6 Institute for Molecular Medicine Finland (FIMM), University of Helsinki, FI, Helsinki, Finland; 7 Division of Cardiology, Azienda Ospedaliero–Universitaria di Parma, Parma, Italy; 8 Associazione per lo Studio Della Trombosi in Cardiologia, Pavia, Italy; 9 Department of Physiology and Biophysics, University of Mississippi Medical Center, Jackson, Mississippi, United States of America; 10 Deutsches Herzzentrum München, Technische Universität München, Deutsches Zentrum für Herz-Kreislauf-Forschung, München, Germany; 11 University of Ottawa Heart Institute, Ottawa, Ontario, Canada; 12 Division of Cardiovascular Medicine, Radcliffe Department of Medicine, University of Oxford, Oxford, United Kingdom; 13 Wellcome Trust Centre for Human Genetics, University of Oxford, Oxford, United Kingdom; 14 Cardiovascular Epidemiology and Genetics, Hospital del Mar Research Institute, Barcelona, Spain; 15 CIBER Enfermedades Cardiovasculares (CIBERCV), Barcelona, Spain; 16 Facultat de Medicina, Universitat de Vic-Central de Cataluña, Vic, Spain; 17 Department of Cardiovascular Sciences, University of Leicester, and NIHR Leicester Biomedical Research Centre, Leicester, United Kingdom; 18 The Zena and Michael A. Wiener Cardiovascular Institute, Icahn School of Medicine at Mount Sinai, New York, New York, United States of America; 19 Institute for Cardiogenetics, University of Lübeck, Lübeck, Germany; 20 DZHK (German Research Centre for Cardiovascular Research), partner site Hamburg/Lübeck/Kiel, Lübeck, Germany; 21 Cardiovascular Epidemiology Unit, Department of Public Health and Primary Care, University of Cambridge, Cambridge, United Kingdom; 22 Wellcome Trust Sanger Institute, Hinxton, Cambridge, United Kingdom; 23 National Institute of Health Research Blood and Transplant; Research Unit in Donor Health and Genomics, University of Cambridge, Cambridge, United Kingdom; 24 Department of Biostatistics and Epidemiology, Perelman School of Medicine, University of Pennsylvania, Philadelphia, Pennsylvania, United States of America; 25 Center for Non-Communicable Diseases, Karachi, Pakistan; 26 Perelman School of Medicine, University of Pennsylvania, Philadelphia, Pennsylvania, United States of America; 27 Veterans Affairs Palo Alto Health Care System, Palo Alto, California, United States of America; 28 Human Genetics Center, School of Public Health, The University of Texas Health Science Center at Houston, Houston, Texas, United States of America; 29 Cardiology Division, Massachusetts General Hospital, Boston, Massachusetts, United States of America; 30 Verve Therapeutics, Boston, Massachusetts, United States of America; Vanderbilt University Medical Center, UNITED STATES

## Abstract

Analyzing 12,361 all-cause cirrhosis cases and 790,095 controls from eight cohorts, we identify a common missense variant in the Mitochondrial Amidoxime Reducing Component 1 gene (*MARC1* p.A165T) that associates with protection from all-cause cirrhosis (OR 0.91, p = 2.3*10^−11^). This same variant also associates with lower levels of hepatic fat on computed tomographic imaging and lower odds of physician-diagnosed fatty liver as well as lower blood levels of alanine transaminase (-0.025 SD, 3.7*10^−43^), alkaline phosphatase (-0.025 SD, 1.2*10^−37^), total cholesterol (-0.030 SD, p = 1.9*10^−36^) and LDL cholesterol (-0.027 SD, p = 5.1*10^−30^) levels. We identified a series of additional *MARC1* alleles (low-frequency missense p.M187K and rare protein-truncating p.R200Ter) that also associated with lower cholesterol levels, liver enzyme levels and reduced risk of cirrhosis (0 cirrhosis cases for 238 R200Ter carriers versus 17,046 cases of cirrhosis among 759,027 non-carriers, p = 0.04) suggesting that deficiency of the MARC1 enzyme may lower blood cholesterol levels and protect against cirrhosis.

## Author summary

Cirrhosis is a leading cause of death worldwide. However, the genetic underpinnings of cirrhosis remain poorly understood. In this study, we analyze twelve thousand individuals with cirrhosis and identify a common missense variant in a gene called *MARC1* that protects against cirrhosis. Carriers of this missense variant also have lower blood cholesterol levels, lower liver enzyme levels and reduced liver fat. We identify an additional two low-frequency coding variants in *MARC1* that are also associated with lower cholesterol levels, lower liver enzyme levels and protection from cirrhosis. Finally, we identify an individual homozygous for a predicted loss-of-function variant in *MARC1* who exhibits very low blood LDL cholesterol levels. These genetic findings suggest that MARC1 deficiency may lower blood cholesterol levels and protect against cirrhosis, pointing to MARC1 as a potential therapeutic target for liver disease.

## Introduction

Discovery of novel protective human genetic variation can identify new therapeutic targets for treatment of a given disease [1]. Targets with human genetic support are more than twice as likely to result in successful development of a therapeutic than targets without genetic support [2]. Indeed, identification of novel protective variants for coronary artery disease and type 2 diabetes, such as variation in *ANGPTL3*, *ANGPTL4*, and *APOC3*, has catalyzed the development of new therapeutics targeting these genes for treatment of metabolic disorders [3–6]. Although liver cirrhosis is a leading cause of death worldwide, no therapies currently exist to treat or delay the progression of cirrhosis [7]. Identification of novel protective variants for cirrhosis may therefore allow for the identification of new therapeutic targets with enhanced likelihood of successful clinical development for treatment of cirrhosis.

Cirrhosis is often considered to be the final stage of distinct pathogenic processes including excess alcohol consumption, viral infection and fatty liver secondary to obesity [8]. However, analyses of these separate processes have identified similar genetic determinants. For example, *PNPLA3* p.I48M and *TM6SF2* p.E40K, although initially identified as associated with hepatic steatosis [9,10], strongly predispose to the development of alcoholic cirrhosis [11], non-alcoholic cirrhosis [12,13] and hepatitis C-related cirrhosis [14,15]. The recently identified splice variant rs72613567 in *HSD17B13* similarly protects against alcoholic cirrhosis, non-alcoholic cirrhosis and severe liver fibrosis among individuals with hepatitis C (**S1 Fig**) [16,17]. These individual findings suggest that analysis of an all-cause cirrhosis phenotype combining alcoholic and non-alcoholic causes may prove useful to find new protective genetic determinants.

In this study, we first examine whether known alcoholic and non-alcoholic cirrhosis variants associate with all-cause cirrhosis. After demonstrating that known alcoholic and non-alcoholic variants associate with all-cause cirrhosis, we leverage the increased power provided by analysis of all-cause cirrhosis to identify a novel common protective missense variant in *MARC1*. We further identify a low-frequency coding variant and a rare stop codon in *MARC1* that form an allelic series associated with lower cholesterol levels, lower liver enzyme levels and protection from cirrhosis.

## Results

### Known alcoholic and non-alcoholic cirrhosis variants associate with all-cause cirrhosis

We first examined whether known alcoholic and non-alcoholic cirrhosis variants associate with all-cause cirrhosis. We created an all-cause liver cirrhosis phenotype in UK Biobank, combining the following ICD10 diagnostic codes: K70.2 (alcoholic fibrosis and sclerosis of the liver), K70.3 (alcoholic cirrhosis of the liver), K70.4 (alcoholic hepatic failure), K74.0 (hepatic fibrosis), K74.1 (hepatic sclerosis), K74.2 (hepatic fibrosis with hepatic sclerosis), K74.6 (other and unspecified cirrhosis of liver), K76.6 (portal hypertension), or I85 (esophageal varices). Using this definition, we identified 1,740 cases of all-cause cirrhosis in UK Biobank. We examined the association of all-cause cirrhosis with six genetic variants previously reported to be associated with alcoholic or non-alcoholic cirrhosis: *PNPLA3* I48M, *TM6SF2* E167K, *MBOAT7* rs641738, *HSD17B13* rs72613567, *HFE* C282Y and *SERPINA1* E366K [11,16,18,19]. All six variants associated with all-cause cirrhosis in UK Biobank (**S2 Fig**). Each variant exhibited greater statistical significance with all-cause cirrhosis than with alcoholic or non-alcoholic subtypes in UK Biobank, with an average 30% gain in power by analyzing all-cause cirrhosis compared to non-alcoholic cirrhosis and an 87% gain in power compared to analyzing alcoholic cirrhosis.

### MARC1 p.A165T associates with protection from cirrhosis, fatty liver, elevated liver enzymes and elevated blood cholesterol levels

**Fig 1:**
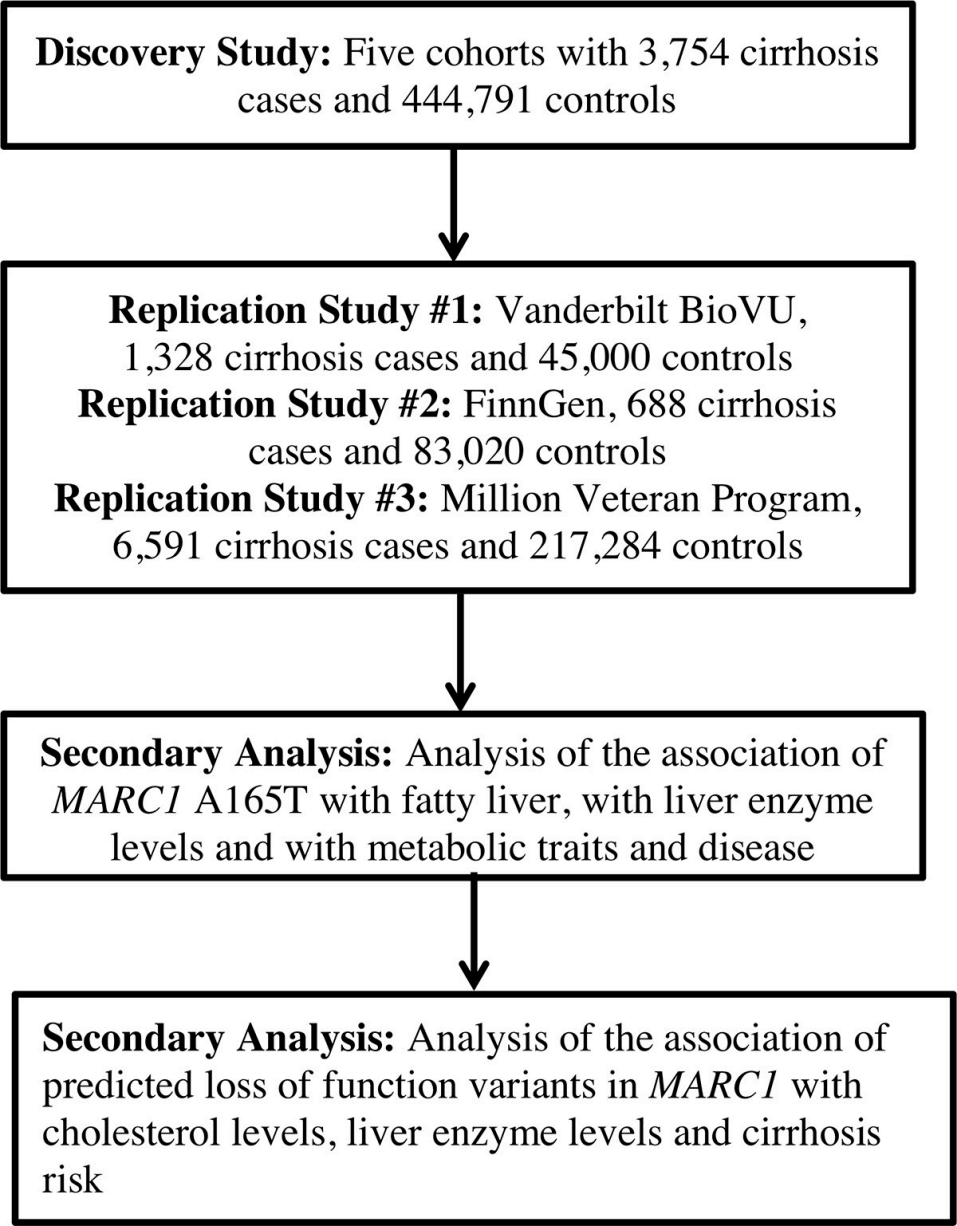
Study design.

Having established that an analysis of all-cause cirrhosis would provide improved statistical power, we sought to identify novel genetic determinants of all-cause cirrhosis through a discovery genome-wide association analysis followed by replication (**Fig 1**). In the discovery analysis, we analyzed 3,754 all-cause cirrhosis cases and 444,791 controls from five cohorts **(S1 Table**). Baseline characteristics and distribution of ancestry in each cohort are provided (**S2 Table, S3 Table**). Mean ages across the studies ranged from 45 years to 57 years. Proportion of female gender was similar in hospital and population-based studies (53.8% to 54.6%), but lower in the alcoholic cirrhosis case-control studies (8.0% and 27.2%). The proportion of individuals with cirrhosis was higher in Partners Biobank (3.9%), a hospital-based cohort, than UK Biobank (0.4%) or ARIC (0.9%), population-based cohorts.

We tested the association of 14 million genetic variants with minor allele frequency > 0.1% with all-cause cirrhosis in both additive and recessive models. No evidence of genomic inflation was observed (lambda 1.02, **S3 Fig**). We replicated known associations of *PNPLA3*, *TM6SF2*, *HFE* and *HDS17B13* variants with cirrhosis at genome-wide significance (**Table 1**). No other variants were associated with all-cause cirrhosis at genome-wide significance.

**Table 1:** DNA sequence variants associated with all-cause cirrhosis in the discovery analysis.

Model	Variant	CHR	EA	EAF	Gene	Annotation	OR	p-value
Additive	rs738409	22	G	26%	*PNPLA3*	Missense: p.I48M	1.47	2.2*10^−67^
Additive	rs58542926	19	T	7%	*TM6SF2*	Missense: p.E167K	1.42	9.7*10^−24^
Recessive	rs1800562	6	A	3%	*HFE*	Missense: p.C282Y	3.2	1.3*10^−14^
Additive	rs72613567	4	TA	22%	*HSD17B13*	Splice Variant	0.82	4.5*10^−8^
Additive	rs2642438	1	A	25%	*MARC1*	Missense: p.A165T	0.87	8.7*10^−7^

CHR: chromosome, EA: effect allele, EAF: effect allele frequency

**Fig 2:**
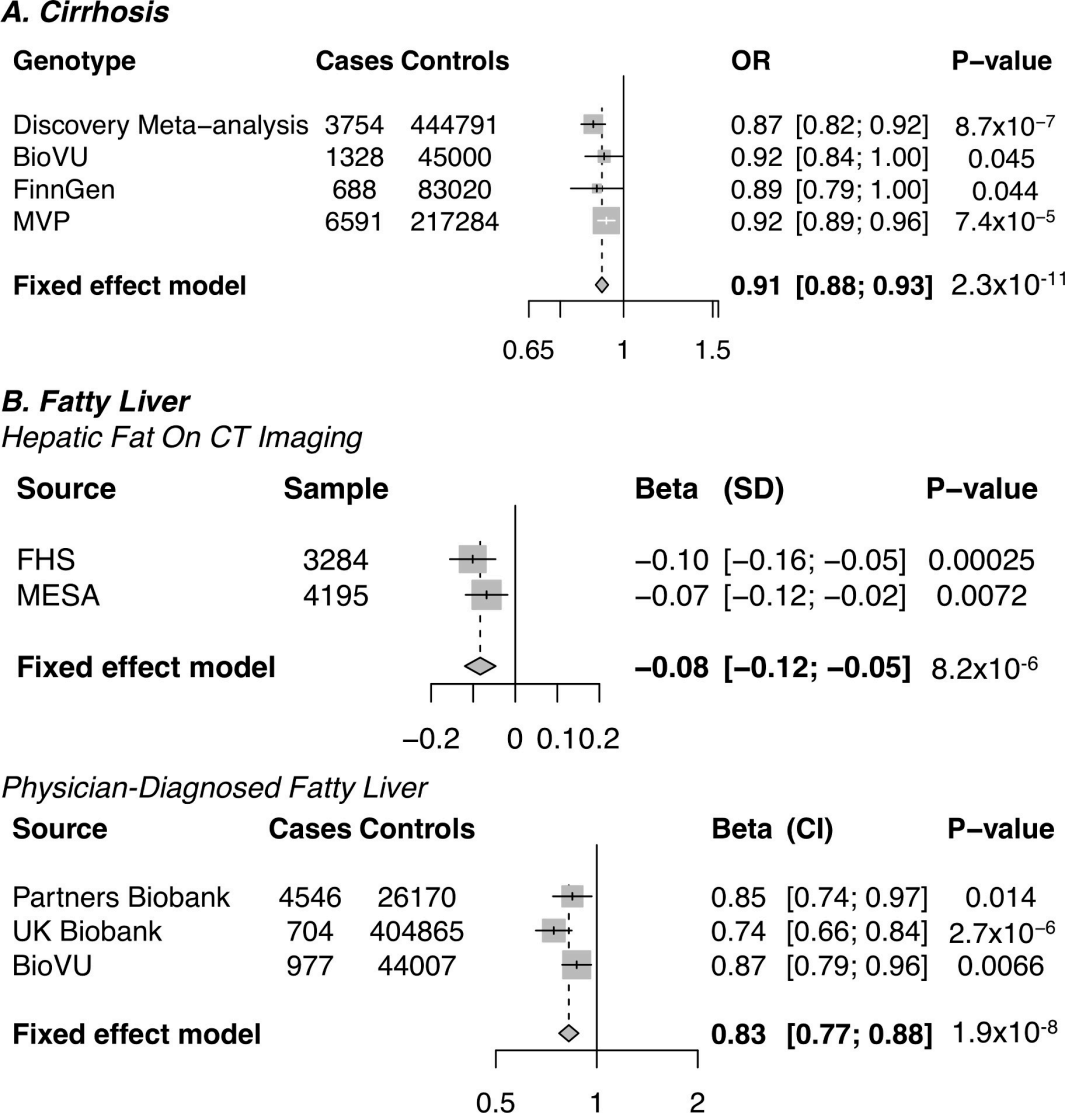
Association of MARC1 p.A165T with cirrhosis and fatty liver in discovery and replication datasets. OR, odds ratio; SD, standard deviation; CI, 95% confidence interval; MVP, Million Veterans Program; FHS, Framingham Heart Study; MESA, Multi-ethnic Study of Atherosclerosis.

The lead coding variant at sub-genome wide significance was a common missense variant in *MARC1* (p.A165T) that was associated with lower risk of all-cause cirrhosis (OR 0.87, p = 8.7*10^−7^, minor allele frequency 25%). We sought replication of this observation in three independent studies: BioVU, FinnGen Consortium and Million Veteran Program. *MARC1* p.A165T associated with protection from cirrhosis in BioVU (OR 0.92, p = 0.045), in FinnGen (0.89, p = 0.044) and in the Million Veteran Program (OR 0.92, p = 7.4*10^−5^). When the statistical evidence from the discovery and replication studies are combined, *MARC1* p.A165T associated with protection from cirrhosis at a significance level exceeding genome wide significance (OR 0.91, p = 2.3*10^−11^, **Fig 2**). No evidence of heterogeneity in the association of *MARC1* p.A165T with all-cause cirrhosis in the discovery analysis and replication analyses was observed (test for heterogeneity p = 0.64). In UK Biobank, *MARC1* p.A165T associated with protection from both alcoholic (OR 0.86, p = 0.05) and non-alcoholic (OR 0.85, p = 0.0002) cirrhosis subtypes. *MARC1* p.A165T also associated with protection in cohorts where cirrhosis was ascertained using ICD codes (OR 0.91, p = 1.9x10^-10^) as well as in cohorts where cirrhosis was ascertained using clinical evaluation (OR 0.88, p = 0.04, p interaction = 0.65, **S4 Fig**).

Next, we examined whether *MARC1* p.A165T associated with fatty liver (definitions provided in **S4 Table**). *MARC1* p.A165T was associated with reduced hepatic fat on computed tomographic imaging in both the Framingham Heart Study (FHS) and Multi-ethnic Study of Atherosclerosis (MESA) cohorts (-0.08 SD, p = 8.2*10^−6^). *MARC1* p.A165T also associated with reduced risk of physician-diagnosed fatty liver in three biobank studies (OR 0.83, p = 1.90*10^−8^).

Having established that *MARC1* p.A165T associates with protection from all-cause cirrhosis as well as fatty liver, we tested association of this variant with plasma biomarkers–ALT (alanine transaminase), AST (aspartate transaminase), ALP (alkaline phosphatase), total cholesterol, LDL (low-density lipoprotein) cholesterol, HDL (high-density lipoprotein) cholesterol, and triglycerides. *MARC1* p.A165T associated with lower ALT levels (-0.025 SD, p = 3.7*10^−43^), AST levels (-0.013 SD, p = 1.8*10^−11^) and ALP levels (-0.025 SD, p = 1.2*10^−37^). *MARC1* p.A165T also associated with lower total cholesterol (-0.030, p = 1.9*10^−36^) and LDL cholesterol (-0.027, p = 5.1*10^−30^). *MARC1* p.A165T associated with higher triglyceride levels (0.013 SD, p = 3.0*10^−9^) and lower HDL cholesterol levels (-0.028 SD, p = 1.3*10^−30^) but did not associate with blood pressure, BMI or waist-to-hip ratio (**S5 Table**).

### MARC1 p.A165T is not associated with coronary artery disease

Variant alleles in *PNPLA3* and *TM6SF2* that decrease risk of cirrhosis have been reported to increase risk for coronary artery disease (CAD) [20]. This raises the possibility that treatment of cirrhosis may have adverse cardiovascular effects. We therefore examined whether *MARC1* p.A165T associates with increased CAD risk. In contrast to *PNPLA3* and *TM6SF2*, neither *MARC1* p.A165T nor *HSD17B13* rs72613567 associated with risk of CAD (**S5 Fig**) [21]. In a phenome wide association study in UK Biobank, *MARC1* p.A165T associated with a lower risk of gallstones (OR 0.96, p = 0.0006) and an elevated risk of gout (OR 1.06, p = 0.001, **S6 Fig**).

### Identification of a series of protective alleles in MARC1

We investigated whether loss or gain of MARC1 function might be responsible for the protection from cirrhosis and the reduced levels of liver enzymes and cholesterol observed. We leveraged a rare nonsense mutation observed early in the *MARC1* gene (p.R200Ter). In a combined analysis of UK Biobank, Partners Biobank, MESA, Framingham, and Million Veteran Program, no cases of cirrhosis were observed among 238 carriers of *MARC1* R200Ter compared to 17,046 cases of cirrhosis among 759,027 non-carriers (odds ratio 0, p = 0.04).

We assembled sequence data for *MARC1* in 45,493 individuals and identified 94 carriers of *MARC1* p.R200Ter and 21 carriers of other predicted loss of function variants (**S6 Table, S7 Table**). Carriers of R200Ter and other loss of function variants in *MARC1* had lower total cholesterol levels (-0.16 SD, p = 0.04) and ALT levels (-0.24 SD, p = 0.02, **Table 2**).

**Table 2:** An allelic series of variants in *MARC1* which associate with lower total cholesterol levels, alanine transaminase levels and reduced risk of cirrhosis.

MARC1 Variant	MAF	Total Cholesterol, Effect in SD (CI)	Alanine Transaminase, Effect in SD (CI)	Cirrhosis, Odds Ratio (CI)
A165T	29%	**-0.030** (-0.035, -0.025), p = 1.9x10^-36^	**-0.025** (-0.028, -0.021) p = 3.7x10^-43^	**0.91** **(**0.88, 0.93), p = 2.3x10^-11^
M187K	1.1%	**-0.053** (-0.074, -0.032), p = 7x10^-7^	**-0.032** (-0.053, -0.012), p = 0.001	**0.75** (0.60, 0.95), p = 0.01
R200Ter	0.009%	**-0.16** (-0.32, -0.01), p = 0.04	-**0.24** (-0.43, -0.05), p = 0.02	**0** (0, 0.90), p = 0.04

Minor allele frequency refers to minor allele frequency in UK Biobank. MAF, minor allele frequency; Dir., direction; SD, standard deviation; OR, odds ratio

R200Ter is enriched among individuals of Ashkenazi Jewish ancestry (0.4% frequency) compared to European ancestry (0.006% frequency). Therefore, in a sensitivity analysis, we matched each R200Ter carrier to the three individuals in UK Biobank who were most similar in ancestry. Carriers of R200Ter had lower ALT levels than non-carriers matched in ancestry (-0.26 SD, p = 0.03). R200Ter similarly associated with lower ALT levels (-0.24 SD, p = 0.04) and total cholesterol levels (-0.23 SD, p = 0.05) when individuals of European and Ashkenazi Jewish ancestry were analyzed separately. In Million Veteran Program, no cases of cirrhosis were observed among 15 R200Ter carriers of European ancestry and no cases of cirrhosis were observed among 6 R200Ter carriers of Ashkenazi Jewish ancestry.

To test whether other coding variants in *MARC1* may influence liver disease risk, we conditioned on A165T and tested whether other variants in *MARC1* associated with ALT levels and cholesterol. We identified a low-frequency missense variant in *MARC1* p.M187K (1.0% frequency in Europeans) that also associated with lower total cholesterol levels (-0.053 SD, 7*10^−7^) and lower ALT levels (-0.032 SD, p = 0.001). This variant also associated with protection from cirrhosis (OR 0.75, p = 0.01). Similar to *MARC1* p.A165T, p.M187K also associated with protection from physician-diagnosed fatty liver (OR 0.76, p = 0.02) and with protection from any liver disease (either cirrhosis or fatty liver, OR 0.75, p = 0.001). *MARC1* p.A165T, p.M187K and p.R200Ter form an allelic series which protect against elevated cholesterol levels, elevated ALT levels and risk of cirrhosis **(Table 2).**

### Identification of an individual homozygous for a predicted loss-of-function variant in MARC1

We wondered if homozygous *MARC1* deficiency might be tolerated in humans. From exome sequencing data in the T2D-GENES consortium, we identified an individual who was homozygous for the loss-of-function variant R200Ter. Although we did not have liver enzyme measurements for this individual, a blood lipid profile was available.

This individual had low LDL cholesterol levels (46 mg/dl, **Table 3**) despite not receiving any lipid lowering therapy. However, the participant had elevated triglyceride levels (375 mg/dl, **Table 3**). These results suggest that homozygous *MARC1* deficiency is both tolerated and results in a lipid phenotype (lower LDL cholesterol levels but higher triglyceride levels) similar to the common A165T variant.

**Table 3:** Blood lipids in an individual homozygous for a predicted loss-of-function variant in *MARC1*. Blood lipids were measured in fasting state.

Genotype	Homozygous for R200Ter
Age	50
Total Cholesterol, mg/dl	159
LDL Cholesterol, mg/dl	46
HDL Cholesterol, mg/dl	38
Triglycerides, mg/dl	375

## Discussion

Here, we identify *MARC1* A165T as a novel genetic determinant of fatty liver and all-cause cirrhosis. We further identify an allelic series of coding variants in *MARC1*, including M187K and R200Ter, which associate with lower liver enzymes levels, lower blood cholesterol levels and protection from liver disease. These findings suggest that therapeutic MARC1 antagonism may be useful for prevention and treatment of liver disease.

*MARC1* encodes Mitochondrial Amidoxime-Reducing Component 1, a molybdenum-containing enzyme [22]. It contains an N-terminal transmembrane helix that anchors the protein to the outer mitochondrial membrane, with the enzymatic domain of MARC1 located in the cytosol [23]. The crystal structure of MARC1 was recently described [24]. The molybdenum cofactor is coordinated in a solvent exposed center by predominantly positively charged amino acids. The A165 residue lies within an alpha helix in the N-terminal domain of MARC1. The function of MARC1 is unknown, however, it has been reported to activate N-hydroxylated prodrugs [25], reduce nitrite to produce nitric oxide [26] and detoxify trimethylamine N-oxide [27].

The mechanism by which *MARC1* may contribute to liver damage and cirrhosis is unclear. The lack of association of *MARC1* p.A165T and *HSD17B1*3 rs72613567 with CAD (in contrast to *PNPLA3* and *TM6SF2*) suggests that pharmacologic treatment of cirrhosis and hepatic steatosis may not universally cause excess cardiovascular risk.

A limitation of the current study is that the cohorts analyzed were overwhelmingly of European ancestry (83% in UK Biobank). Analysis of trans-ethnic cohorts may clarify whether variation in MARC1 contributes to cirrhosis protection among individuals of non-European ancestry. Analysis of biopsy-diagnosed samples may also clarify whether, like *HSD17B13* [16,17], *MARC1* has differing associations with fatty liver, non-alcoholic steatohepatitis and cirrhosis. Additionally, in this report, we do not provide any biochemical or functional evidence linking MARC1 to liver disease; such studies are warranted and underway.

Despite the substantial burden of disease posed by cirrhosis worldwide [28], identification of genetic risk factors has been limited relative to other common diseases such as type 2 diabetes, CAD or inflammatory bowel disease. Here we show that joint analysis of alcoholic and non-alcoholic cirrhosis cases from multiple cohorts increases statistical power to identify genetic variants that influence cirrhosis, to identify novel therapeutic targets and to further our understanding of this disease.

## Methods

### Association of known alcoholic and non-alcoholic cirrhosis variants with all-cause cirrhosis in UK Biobank

To examine whether known alcoholic and non-alcoholic cirrhosis variants associate with all-cause cirrhosis, we tested the association of six known cirrhosis variants (*PNPLA3* I48M, *TM6SF2* E167K, *MBOAT7* rs641738, *HSD17B13* rs72613567, *HFE* C282Y and *SERPINA1* E366K[11,16,18,19]) with all-cause cirrhosis in UK Biobank (hospitalization or death due to ICD codes K70.2, K70.3, K70.4, K74.0, K74.1, K74.2, K74.6, K76.6, or I85). To examine whether this approach increased power relative to examining subtypes of alcoholic and non-alcoholic cirrhosis, we compared the significance of the association of these variants with all-cause cirrhosis (their Z-scores) to the significance of the association of these variants separately with alcoholic cirrhosis and with non-alcoholic cirrhosis. Alcoholic cirrhosis was defined as physician-diagnosed alcoholic cirrhosis or alcoholic liver failure (ICD codes K70.2, K70.3 or K70.4). Non-alcoholic cirrhosis was defined as non-alcoholic cirrhosis (ICD codes K74.0, K74.1, K74.2, K74.6, K76.6 or I85) that occurred among individuals who drank less than fourteen alcoholic drinks per week. We excluded former drinkers (individuals who previously consumed alcohol but stopped) from analysis of non-alcoholic cirrhosis, as these individuals may have previously consumed alcohol but quit due to adverse effects [29]. We tested the association of each of the six variants with all-cause cirrhosis, alcoholic cirrhosis and non-alcoholic cirrhosis in UK Biobank using logistic regression adjusted for age, sex, ten principal components of ancestry and a dummy variable for array type.

### Genome wide association study for all-cause cirrhosis

We conducted a genome wide association for all-cause cirrhosis using five cohorts: UK Biobank, Partners Biobank, Atherosclerosis Risk in Communities study (ARIC) and summary statistics from two cohorts from a prior genome wide association study of alcoholic cirrhosis [11]. Definitions of cirrhosis used in each of the five cohorts are provided (**S1 Table**). We excluded cases of cirrhosis secondary to primary biliary cholangitis and primary sclerosis cholangitis as these autoimmune disorders are directed against the biliary (and not hepatic) parenchyma [30,31].

For UK Biobank, genotyping was performed using either the UK BiLEVE Axiom array or the UK Biobank Axiom array. Phasing and imputation were performed centrally, by UK Biobank, using the Haplotype Reference Consortium and a reference panel of UK 10K merged with the 1000 Genomes phase 3 panel. One related individual of each related pair of individuals, individuals whose genetic sex did not match self-reported sex and individuals with an excess of missing genotype calls or more heterozygosity than expected were excluded from analysis. For Partners Biobank, genotyping was performed using Illumina MEGA array. Variants were imputed to the HapRef consortium using the Michigan Imputation Server [32]. For ARIC, genotyping was performed using the Affymetrix 6.0 array. Variants were imputed to the HapRef consortium using the Michigan Imputation Server. We excluded any variants with an imputation quality < 0.3.

Genome wide association study in each cohort was performed using logistic regression with adjustment for age, sex and principal components of ancestry. In UK Biobank, Partners Biobank, ARIC, BioVU and FinnGen, ancestry was controlled for through inclusion of ten principal component of ancestry covariates. In MVP, ancestry was controlled for through inclusion of five principal component of ancestry covariates. In the AlcCir consortium case-control studies, ancestry was adjusted for by restricting analysis to White British and German populations. We tested the association of fourteen million variants with minor allele frequency of greater than 0.1% with cirrhosis in each cohort. PLINK was used for all analyses [33]. To combine estimates across cohorts, inverse variance fixed effects meta-analysis, as implemented by METAL, was used [34]. Quantile-quantile analysis was used to examine for the presence of population stratification. No evidence of inflation was observed (lambda 1.02; **S2 Fig**). Both additive and recessive analyses were performed.

### Replication of the association of MARC1 p.A165T with cirrhosis and fatty liver

We replicated the association of *MARC1* p.A165T with all-cause cirrhosis in three cohorts. First, we examined whether *MARC1* p.A165T associates with physician-diagnosed all-cause cirrhosis in the Vanderbilt BioVU, a DNA databank linked to de-identified electronic health records. We identified 46,328 individuals of European ancestry with genome-wide genotyping and who were either cases or controls for all-cause cirrhosis. Using hospitalization or death due to ICD-9 (571.2, 571.5, 572.3, 456.0, 456.1, 456.2) or ICD-10 codes (K70.2, K70.3, K70.4, K74.0, K74.1, K74.2, K74.6, K76.6, I85) to define all-cause cirrhosis, 1,328 cases were identified. We identified 45,000 controls using the EHR-based PheWAS approach, which excludes related diseases based on ICD codes [35]. Logistic regression, with adjustment for age, sex and principal components of ancestry, was used to estimate the association of *MARC1* p.A165T with cirrhosis in this dataset. Second, in FinnGen, we identified 688 cases of all-cause cirrhosis (ICD-10 K70.2, K70.3, K70.4, K74.0, K74.1, K74.2, K74.6, K76.6, I85) and 83020 controls. Logistic regression, as implemented in SAIGE [36], was used to test the association of *MARC1* p.A165T with cirrhosis in this dataset while controlling for age, sex and relatedness within the sample. Third, in the Million Veteran Program, we identified 6,591 cases of all-cause cirrhosis (as defined above) and 217,284 controls among individuals of European ancestry. Logistic regression was also used to estimate the association of *MARC1* p.A165T with cirrhosis in this dataset, with adjustment for age, sex and five principal components of ancestry.

To examine whether *MARC1* p.A165T associates with fatty liver, we tested the association of this variant with fatty liver in five cohorts. In the Framingham cohort (Offspring Cohort and Third Generation Cohort), we examined whether *MARC1* p.A165T associates with hepatic steatosis on CT imaging. 3284 individuals in Framingham with genotype data available underwent multidetector abdominal CT [37]. We measured hepatic steatosis by computing the liver-to-phantom ratio of the average Hounsfield units of three liver measurements to average Hounsfield units of three phantom measurements (to correct for inter-individual differences in penetration), as previously described [37]. We tested the association of the p.A165T variant with liver-to-phantom ratio with adjustment for age, sex and ten principal components of ancestry using a linear mixed model to control for relatedness among individuals. The liver-to-phantom ratio was subject to inverse normal transformation prior to analysis. In the Multi-Ethnic Study of Atherosclerosis cohort (MESA), 4195 individuals underwent multidetector CT. Hepatic steatosis was measured as the mean of three attenuation measurements (two in the right lobe of the liver and one in the left lobe). No phantom measurement was available for standardization. We therefore tested the association of the p.A165T variant with mean liver attenuation with adjustment for age, sex and ten principal components of ancestry. Mean liver attenuation was subject to inverse normal transformation prior to analysis. Individuals with higher liver fat have *lower* liver-to-phantom ratios and liver attenuation measurements. For interpretability, we therefore report all estimates in units of standard deviation increases in liver fat, with a one standard deviation increase in liver fat corresponding to a one standard deviation decrease in the liver-to-phantom ratio or mean liver attenuation.

In three cohorts (Partners Biobank, UK Biobank, BioVU), we lacked CT imaging data to measure hepatic steatosis. We therefore tested the association of the *MARC1* p.A165T variant with physician-diagnosed fatty liver in these cohorts (ICD codes K76.0 fatty change of liver, K75.81 non-alcoholic steatohepatitis) using logistic regression, adjusted for age, sex and ten principal components of ancestry. We pooled estimates of the association of *MARC1* p.A165T with fatty liver across all five cohorts using fixed effects meta-analysis [34].

### Association of MARC1 p.A165T with liver enzyme levels, metabolic traits and disease

We tested the association of p.A165T with metabolic traits using four different datasets. For serum levels of liver enzymes, we used data from UK Biobank (n = 386273), Partners Biobank (n = 26,471), Framingham (n = 3,288), LOLIPOP (n = 54,857) [38], BioBank Japan (n = 134,182) [39] where measures of serum alanine transaminase (ALT), aspartate transaminase (AST) and alkaline phosphatase (ALP) were available. We log transformed ALT, AST and ALP. We then conducted a linear regression analysis with adjustment for age, sex and ten principal components of ancestry. We pooled estimates across cohorts using inverse variance weighted fixed effects meta-analysis. For lipids [low-density lipoprotein (LDL) cholesterol, high-density lipoprotein (HDL) cholesterol, triglycerides and total cholesterol], we used data from UK Biobank (n = 386,435) and the Global Lipids Genetics Consortium, a meta-analysis of 188 587 individuals of European descent [40]. This GWAS included 37 studies genotyped using the Illumina Metabochip array as well as an additional 23 studies genotyped using a variety of arrays. For BMI and WHRadjBMI we used data from the Genetic Investigation of ANthropometric Traits (GIANT) consortium [41,42]. For WHRadjBMI, data from 210,088 individuals of European ancestry were included. For BMI, data for 322,154 individuals of European ancestry were included. Individuals were genotyped using various arrays and imputed with the HapMap reference panel to 2.5 million SNPs. For blood pressure, we used data from UK Biobank. We tested the association of p.A165T with systolic blood pressure and diastolic blood pressure using linear regression with adjustment for age, sex and ten principal components of ancestry.

A phenome-wide association study of *MARC1* p.A165T in UK Biobank was performed [43,44]. We tested the association of *MARC1* p.A165T with diseases with more than one thousand cases in UK Biobank. Definitions for 31 different diseases analyzed in the phenome-wide association study are provided (**S8 Table**). The association of p.A165T with each disease was estimated using logistic regression with adjustment for age, sex, ten principal components of ancestry and a dummy variable for array type. A Bonferroni adjusted significance level of p < 0.0016 (0.05/31) was used.

### Association of a rare stop codon in MARC1 with cholesterol levels, liver enzyme levels and cirrhosis

*MARC1* p.A165T associated with lower total cholesterol levels, lower alanine transaminase levels and reduced risk of cirrhosis. To examine whether *MARC1* deficiency may therefore protect against these three phenotypes, we leveraged a rare (frequency 0.009%) stop codon in *MARC1* (Arg200Ter, rs139321832). This stop codon truncates the enzymatic domain of *MARC1* before the catalytic site [24]. We tested the association of this rare stop variant with three phenotypes.

First, we examined the association of this stop codon in *MARC1* with total cholesterol using data from the Global Lipids Genetics Consortium exome chip analysis (n = 283474) and UK Biobank (n = 386435) [20]. We also included additional rare predicted loss of function variants from two exome sequence datasets: the Myocardial Infarction Genetics consortium (n = 27034) and the T2D Genes consortium (n = 18456). Sequence data for *MARC1* were extracted from exome sequencing performed in the MIGen Consortium and T2D Genes consortiums as previously described [45,46]. Estimates were adjusted for age, sex and five principle components of ancestry. We pooled estimates from all three data sources using inverse variance weighted fixed effects meta-analysis.

Second, we examined the association of the rare stop codon with ALT levels using data from UK Biobank and the Partners Biobank cohort. We tested for the association of this variant with inverse normal transformed ALT levels using linear regression with adjustment for age, sex and ten principal components of ancestry.

Third, we examined the association of the rare stop codon with cirrhosis risk using data from UK Biobank, Partners Biobank and Million Veteran Program. Odds ratios for cirrhosis were pooled across cohorts using meta-analysis. As this variant is more frequently observed in Ashkenazi Jewish ancestry than individuals of European ancestry, we examined whether this variant also associates with liver traits with individuals of Ashkenazi Jewish ancestry excluded. Individuals with Ashkenazi Jewish ancestry were identified using principal component analysis, as previously performed [47]. Self-reported Ashkenazi ancestry was plotted against all ten principal components in UK Biobank. As previously reported, self-reported Ashkenazi Jewish ancestry was found to segregate with principal component four. All individuals in this Ashkenazi Jewish cluster were excluded from analysis in a sensitivity analysis. We also examined whether this rare stop variant associated with protection from liver disease when each individual who carried it was matched to three individuals with the most similar principal components of ancestry.

Fourth, we examined whether homozygosity for predicted loss-of-function variants, as defined above, was tolerated. In UK Biobank, the Myocardial Infarction Genetics consortium and the T2D Genes Consortium, we examined the genotype of all loss-of-function variant carriers to determine if they were homozygous for a loss-of-function variant. For any homozygous individual, we examined if blood lipids, available in all three cohorts, differed from the corresponding general population.

### Ethics statement

This research has been conducted using the UK Biobank resource and was approved by the UK Biobank application committee, application 7089.

## Supporting information

S1 Table Definition of cirrhosis in each cohort. (DOCX)

S2 Table Baseline characteristics of individuals in discovery cohorts. (DOCX)

S3 Table Distribution of ancestry in each cohort. (DOCX)

S4 Table Definition of fatty liver in each cohort. (DOCX)

S5 Table Association of MARC1 A165T with metabolic traits. (DOCX)

S6 Table Rare predicted loss of function variants in MIGEN. (DOCX)

S7 Table Rare predicted loss of function variants in T2D Genes. (DOCX)

S8 Table Definition of outcomes in phenome wide association study in UK Biobank. (DOCX)

S1 Fig Risk of alcoholic, non-alcoholic and hepatitis C cirrhosis associated with PNPLA3 I48M, TM6SF2 E40K and HSD17B13. (PDF)

S2 Fig Association of known alcoholic and non-alcoholic cirrhosis variants with all-cause cirrhosis in UK Biobank. (PDF)

S3 Fig QQ plot for genome wide analysis of cirrhosis. (PDF)

S4 Fig Risk of cirrhosis associated with *MARC1* A165T among cohorts by method of cirrhosis ascertainment. (PDF)

S5 Fig Association of cirrhosis variants with type 2 diabetes, coronary artery disease and cirrhosis. (PDF)

S6 Fig Association of *MARC1* A165T with other diseases in a phenome wide association study. (PDF)
