# Correction: A missense variant in Mitochondrial Amidoxime Reducing Component 1 gene and protection against liver disease

**DOI:** 10.1371/journal.pgen.1009503

**Published:** 2021-04-06

**Authors:** Connor A. Emdin, Mary E. Haas, Amit V. Khera, Krishna Aragam, Mark Chaffin, Derek Klarin, George Hindy, Lan Jiang, Wei-Qi Wei, Qiping Feng, Juha Karjalainen, Aki Havulinna, Tuomo Kiiskinen, Alexander Bick, Diego Ardissino, James G. Wilson, Heribert Schunkert, Ruth McPherson, Hugh Watkins, Roberto Elosua, Matthew J. Bown, Nilesh J. Samani, Usman Baber, Jeanette Erdmann, Namrata Gupta, John Danesh, Danish Saleheen, Kyong-Mi Chang, Marijana Vujkovic, Ben Voight, Scott Damrauer, Julie Lynch, David Kaplan, Marina Serper, Philip Tsao, Million Veteran Program, Josep Mercader, Craig Hanis, Mark Daly, Joshua Denny, Stacey Gabriel, Sekar Kathiresan

In this article, the genetic variant *PNPLA3* p.I148M is misreported in several sentences as *PNPLA3* p.I48M. The variant *TM6SF2* p.E167K is misreported in one sentence as *TM6SF2* p.E40K.

In the Introduction, the third sentence of the second paragraph:

For example, *PNPLA3* p.I48M and *TM6SF2* p.E40K, although initially identified as associated with hepatic steatosis [9,10], strongly predispose to the development of alcoholic cirrhosis [11], non-alcoholic cirrhosis [12,13] and hepatitis C-related cirrhosis [14,15].

should read:

For example, *PNPLA3* p.I1148M and *TM6SF2* p.E167K, although initially identified as associated with hepatic steatosis [9,10], strongly predispose to the development of alcoholic cirrhosis [11], non-alcoholic cirrhosis [12,13] and hepatitis C-related cirrhosis [14,15].

In the Results, the fourth sentence of the first paragraph:

We examined the association of all-cause cirrhosis with six genetic variants previously reported to be associated with alcoholic or non-alcoholic cirrhosis: *PNPLA3* I48M, *TM6SF2* E167K, *MBOAT7* rs641738, *HSD17B13* rs72613567, *HFE* C282Y and *SERPINA1* E366K [11,16,18,19].

should read:

We examined the association of all-cause cirrhosis with six genetic variants previously reported to be associated with alcoholic or non-alcoholic cirrhosis: *PNPLA3* I148M, *TM6SF2* E167K, *MBOAT7* rs641738, *HSD17B13* rs72613567, *HFE* C282Y and *SERPINA1* E366K [11,16,18,19].

In the Methods, the first sentence of the first paragraph:

To examine whether known alcoholic and non-alcoholic cirrhosis variants associate with all-cause cirrhosis, we tested the association of six known cirrhosis variants (*PNPLA3* I48M, *TM6SF2* E167K, *MBOAT7* rs641738, *HSD17B13* rs72613567, *HFE* C282Y and *SERPINA1* E366K [11,16,18,19]) with all-cause cirrhosis in UK Biobank (hospitalization or death due to ICD codes K70.2, K70.3, K70.4, K74.0, K74.1, K74.2, K74.6, K76.6, or I85).

should read:

To examine whether known alcoholic and non-alcoholic cirrhosis variants associate with all-cause cirrhosis, we tested the association of six known cirrhosis variants (*PNPLA3* I148M, *TM6SF2* E167K, *MBOAT7* rs641738, *HSD17B13* rs72613567, *HFE* C282Y and *SERPINA1* E366K [11,16,18,19]) with all-cause cirrhosis in UK Biobank (hospitalization or death due to ICD codes K70.2, K70.3, K70.4, K74.0, K74.1, K74.2, K74.6, K76.6, or I85).

There is an error in Table 1. The name p.I48M appears instead of p.I148M.

**Table 1:** DNA sequence variants associated with all-cause cirrhosis in the discovery analysis.

Model	Variant	CHR	EA	EAF	Gene	Annotation	OR	p-value
Additive	rs738409	22	G	26%	*PNPLA3*	Missense: p.I148M	1.47	2.2*10^−67^
Additive	rs58542926	19	T	7%	*TM6SF2*	Missense: p.E167K	1.42	9.7*10^−24^
Recessive	rs1800562	6	A	3%	*HFE*	Missense: p.C282Y	3.2	1.3*10^−14^
Additive	rs72613567	4	TA	22%	*HSD17B13*	Splice Variant	0.82	4.5*10^−8^
Additive	rs2642438	1	A	25%	*MARC1*	Missense: p.A165T	0.87	8.7*10^−7^

CHR: chromosome, EA: effect allele, EAF: effect allele frequency

There is an error in the label of S1 Fig. The name p.I48M appears instead of p.I148M. The authors have provided a corrected version here.

## Supporting information

S1 Fig Risk of alcoholic, non-alcoholic and hepatitis C cirrhosis associated with PNPLA3 I148M, TM6SF2 E167K and HSD17B13. (PDF)
