# Supplementary material for: A missense variant in Mitochondrial Amidoxime Reducing Component 1 gene and protection against liver disease
Source: PLoS Genet. 2020 Apr 13;16(4):e1008629. doi: 10.1371/journal.pgen.1008629 (PMC7200007; doi:10.1371/journal.pgen.1008629)
Supplement: S1 Table — (DOCX) [file pgen.1008629.s001.docx]

Supplementary Table 1. Definition of cirrhosis in each cohort.

| Cohort | Definition of cirrhosis | Cases | Controls | Individual-level data |
| --- | --- | --- | --- | --- |
| UK Biobank | Hospitalization or death due to physician diagnosed cirrhosis: K70.2 (alcoholic fibrosis and sclerosis of the liver), K70.3 (alcoholic cirrhosis), K70.4 (alcoholic hepatic failure), K74.0 (hepatic fibrosis), K74.1 (hepatic sclerosis), K74.2 (hepatic fibrosis with hepatic sclerosis), K74.6 (other and unspecific cirrhosis of liver), K76.6 (portal hypertension), or I85 (esophageal varices) | 1740 | 403829 | Yes |
| Partners Biobank | Hospitalization or death due to physician diagnosed cirrhosis: K70.2 (alcoholic fibrosis and sclerosis of the liver), K70.3 (alcoholic cirrhosis), K70.4 (alcoholic hepatic failure), K74.0 (hepatic fibrosis), K74.1 (hepatic sclerosis), K74.2 (hepatic fibrosis with hepatic sclerosis), K74.6 (other and unspecific cirrhosis of liver), K76.6 (portal hypertension), or I85 (esophageal varices) | 1214 | 29502 | Yes |
| ARIC | Hospitalization or death due to physician diagnosed cirrhosis: K70.2 (alcoholic fibrosis and sclerosis of the liver), K70.3 (alcoholic cirrhosis), K70.4 (alcoholic hepatic failure), K74.0 (hepatic fibrosis), K74.1 (hepatic sclerosis), K74.2 (hepatic fibrosis with hepatic sclerosis), K74.6 (other and unspecific cirrhosis of liver), K76.6 (portal hypertension), or I85 (esophageal varices) | 88 | 10034 | Yes |
| Alcoholic cirrhosis GWAS: German Cohort | Presence of cirrhosis on liver biopsy (fibrosis stage 5 or 6) or unequivocal clinical and laboratory evidence for the presence of cirrhosis | 410 | 1080 | No |
| Alcoholic cirrhosis GWAS: UK Cohort | Histological examination of liver tissue or compatible historical, clinical, laboratory, radiological and endoscopic features | 302 | 346 | No |
| BioVU | Hospitalization or death due to physician diagnosed cirrhosis: K70.2 (alcoholic fibrosis and sclerosis of the liver), K70.3 (alcoholic cirrhosis), K70.4 (alcoholic hepatic failure), K74.0 (hepatic fibrosis), K74.1 (hepatic sclerosis), K74.2 (hepatic fibrosis with hepatic sclerosis), K74.6 (other and unspecific cirrhosis of liver), K76.6 (portal hypertension), or I85 (esophageal varices) | 1328 | 45000 | Yes |
| FinnGen | Hospitalization or death due to physician diagnosed cirrhosis: K70.2 (alcoholic fibrosis and sclerosis of the liver), K70.3 (alcoholic cirrhosis), K70.4 (alcoholic hepatic failure), K74.0 (hepatic fibrosis), K74.1 (hepatic sclerosis), K74.2 (hepatic fibrosis with hepatic sclerosis), K74.6 (other and unspecific cirrhosis of liver), K76.6 (portal hypertension), or I85 (esophageal varices) | 688 | 83020 | Yes |
| Million Veterans Program | Hospitalization or death due to physician diagnosed cirrhosis: K70.2 (alcoholic fibrosis and sclerosis of the liver), K70.3 (alcoholic cirrhosis), K70.4 (alcoholic hepatic failure), K74.0 (hepatic fibrosis), K74.1 (hepatic sclerosis), K74.2 (hepatic fibrosis with hepatic sclerosis), K74.6 (other and unspecific cirrhosis of liver), K76.6 (portal hypertension), or I85 (esophageal varices) | 6591 | 217284 | Yes |
| Total |  | 12361 | 790,095 |  |
