# Supplementary material for: A missense variant in Mitochondrial Amidoxime Reducing Component 1 gene and protection against liver disease
Source: PLoS Genet. 2020 Apr 13;16(4):e1008629. doi: 10.1371/journal.pgen.1008629 (PMC7200007; doi:10.1371/journal.pgen.1008629)
Supplement: S2 Table — (DOCX) [file pgen.1008629.s002.docx]

Supplementary Table 2. Baseline characteristics of individuals in discovery cohorts.

|  | UK Biobank,  N=405,569 | Partners Biobank,  N=30,716 | Atherosclerosis Risk in Communities, N=10,122 | AlcCir Consortium, German cohort, N=1,490 | AlcCir Consortium, UK Cohort, N=648 |
| --- | --- | --- | --- | --- | --- |
| Age | 57.0 (8.1) | 57.2 (17.2) | 54.2 (5.7) | 45.0 (17.0) | 50.9 (18.3) |
| Female Gender, n (%) | 218376 (53.8%) | 16781 (54.6%) | 5501 (54.3%) | 119 (8.0%) | 176 (27.2%) |
| Body-mass index | 27.4 (4.8) | 28.7 (9.3) | 27.5 (5.2) | 25.2 (7.2) | 24.7 (5.3) |
| Type 2 diabetes, n (%) | 20458 (5.0%) | 3398 (11.1%) | 909 (8.9%) | 142 (9.5%) | 0, (0%) |
| Cirrhosis, n (%) | 1740 (0.4%) | 1214 (3.9%) | 88 (0.9%) | 410 (27.5%) | 302 (46.7%) |

All characteristics are in terms of mean (SD), unless otherwise specified. Median age and body mass index are reported for the AlcCir case-control cohorts^6^, as individual level data for these cohorts was not available. The much higher cirrhosis percentage in the AlcCir case-controls cohorts is caused by the case-control design of these cohorts.
