# Supplementary material for: A missense variant in Mitochondrial Amidoxime Reducing Component 1 gene and protection against liver disease
Source: PLoS Genet. 2020 Apr 13;16(4):e1008629. doi: 10.1371/journal.pgen.1008629 (PMC7200007; doi:10.1371/journal.pgen.1008629)
Supplement: S3 Table — (DOCX) [file pgen.1008629.s003.docx]

Supplementary Table 3. Distribution of ancestry in each cohort.

| Ancestry | UK Biobank,  N=405,569 | Partners Biobank,  N=30,716 | Atherosclerosis Risk in Communities, N=10,122 | AlcCir Consortium, German cohort, N=1,490 | AlcCir Consortium, UK Cohort, N=648 | BioVU, N=46328 | FinnGen,N=83,708 | MVP, N=223,875 |
| --- | --- | --- | --- | --- | --- | --- | --- | --- |
| European | 335,660 (83%) | 25,652 (84%) | 8015 (79%) | 1490 (100%) | 648 (100%) | 46328 (100%) | 83,708 (100%) | 223,875 (100%) |
| African | 7,004 (1.7%) | 1,544 (5%) | 2107 (21%) | 0 (0%) | 0 (0%) | 0 (0%) | 0 (0%) | 0 (0%) |
| Asian | 7,004 (1.7%) | 672 (2%) | 0 (0%) | 0 (0%) | 0 (0%) | 0 (0%) | 0 (0%) | 0 (0%) |
| Other | 55,901 (13.7%) | 2,848 (9%) | 0 (0%) | 0 (0%) | 0 (0%) | 0 (0%) | 0 (0%) | 0 (0%) |
