# Supplementary material for: A missense variant in Mitochondrial Amidoxime Reducing Component 1 gene and protection against liver disease
Source: PLoS Genet. 2020 Apr 13;16(4):e1008629. doi: 10.1371/journal.pgen.1008629 (PMC7200007; doi:10.1371/journal.pgen.1008629)
Supplement: S4 Table — (DOCX) [file pgen.1008629.s004.docx]

Supplementary Table 4. Definition of fatty liver in each cohort.

| Cohort | Definition of cirrhosis | Cases | Controls | Individual-level data |
| --- | --- | --- | --- | --- |
| Framingham | CT: Ratio of mean of liver attenuation measurements to phantom measurement | 3284 | | Yes |
| MESA | CT: Mean of three liver attenuation measurements | 4195 | | Yes |
| UK Biobank | Physician diagnosed: K76.0 (fatty change of liver), K76.5 (non-alcoholic steatohepatitis) | 704 | 404865 | Yes |
| Partners Biobank | Physician diagnosed: K76.0 (fatty change of liver), K76.5 (non-alcoholic steatohepatitis) | 4546 | 26170 | Yes |
| BioVU | Physician diagnosed: K76.0 (fatty change of liver), K76.5 (non-alcoholic steatohepatitis) | 977 | 44007 | Yes |
| Total |  | 488748 | |  |
