# Supplementary material for: A missense variant in Mitochondrial Amidoxime Reducing Component 1 gene and protection against liver disease
Source: PLoS Genet. 2020 Apr 13;16(4):e1008629. doi: 10.1371/journal.pgen.1008629 (PMC7200007; doi:10.1371/journal.pgen.1008629)
Supplement: S5 Table — (DOCX) [file pgen.1008629.s005.docx]

Supplementary Table 5. Association of MARC1 A165T with metabolic traits.

| ***Outcome*** | ***Source*** | ***n*** | ***Beta*** | ***SE*** | ***p-value*** |
| --- | --- | --- | --- | --- | --- |
| *Liver enzymes* |  |  |  |  |  |
| ALT | Partners Biobank, Framingham, LOLIPOP, BioBank Japan, UK Biobank | 607935 | -0.025 | 0.002 | 3.7*10^-43 |
| AST | Partners Biobank, Framingham, LOLIPOP, BioBank Japan, UK Biobank | 606638 | -0.013 | 0.002 | 1.8*10^-11 |
| ALP | Partners Biobank, Framingham, LOLIPOP, BioBank Japan, UK Biobank | 551105 | -0.025 | 0.002 | 1.2*10^-37 |
| *Blood lipids* |  |  |  |  |  |
| Total Cholesterol | GLGC, UK Biobank | 575012 | -0.030 SD | 0.002 | 1.9*10^-36 |
| LDL Cholesterol | GLGC, UK Biobank | 574283 | -0.027 SD | 0.002 | 5.1*10^-30 |
| HDL Cholesterol | GLGC, UK Biobank | 542407 | -0.028 SD | 0.002 | 1.3*10^-30 |
| Triglycerides | GLGC, UK Biobank | 574708 | 0.013 SD | 0.002 | 3.0*10^-9 |
| *Blood pressure* |  |  |  |  |  |
| Systolic blood pressure | UK Biobank | 379771 | 0.001 SD | 0.002 | 0.55 |
| Diastolic blood pressure | UK Biobank | 379782 | 0.003 SD | 0.003 | 0.19 |
| *Anthropometric measurements* |  |  |  |  |  |
| Body mass index | GIANT | 325053 | 0.005 SD | 0.003 | 0.17 |
| Waist to hip ratio adjusted for body mass index | GIANT | 211816 | 0.006 SD | 0.004 | 0.15 |

GLGC: Global lipids genetics consortium, GIANT: Genetic investigation of anthropometric traits consortium, SD: standard deviations
