# Supplementary material for: A missense variant in Mitochondrial Amidoxime Reducing Component 1 gene and protection against liver disease
Source: PLoS Genet. 2020 Apr 13;16(4):e1008629. doi: 10.1371/journal.pgen.1008629 (PMC7200007; doi:10.1371/journal.pgen.1008629)
Supplement: S6 Table — (DOCX) [file pgen.1008629.s006.docx]

Supplementary Table 6. Rare predicted loss of function variants in MIGEN

| **CHR:POS_REF/ALT** | **Consequence** | **Amino Acid Change** | **Individuals With Variant** |
| --- | --- | --- | --- |
| 1:220970148_G/A | Splice Donor |  | 2 |
| 1:220970148_G/C | Splice Donor |  | 1 |
| 1:220971357_G/A | Splice Donor |  | 1 |
| 1:220978456_G/A | Splice Donor |  | 2 |
| 1:220978457_T/C | Splice Donor |  | 1 |
| 1:220986659_C/T | Stop Gained | Arg305Ter | 2 |
| 1:220986755_C/T | Stop Gained | Gln337Ter | 3 |
| Total |  |  | 12 |
