# Supplementary material for: A missense variant in Mitochondrial Amidoxime Reducing Component 1 gene and protection against liver disease
Source: PLoS Genet. 2020 Apr 13;16(4):e1008629. doi: 10.1371/journal.pgen.1008629 (PMC7200007; doi:10.1371/journal.pgen.1008629)
Supplement: S7 Table — (DOCX) [file pgen.1008629.s007.docx]

Supplementary Table 7. Rare predicted loss of function variants in T2D Genes.

| **CHR:POS_REF/ALT** | **Consequence** | **Amino Acid Change** | **Individuals With Variant** |
| --- | --- | --- | --- |
| 1:220960469_TG/T | Frameshift | Trp62fs | 2 |
| 1:220960562_G/A | Splice Donor |  | 1 |
| 1:220970097_C/T | Stop Gained | Arg188Ter | 1 |
| 1:220978402_G/A | Stop Gained | Trp254Ter | 2 |
| 1:220986659_C/T | Stop Gained | Arg305Ter | 2 |
| 1:220986728_TG/T | Frameshift | Val328fs | 1 |
| Total |  |  | 9 |
