# Supplementary material for: A missense variant in Mitochondrial Amidoxime Reducing Component 1 gene and protection against liver disease
Source: PLoS Genet. 2020 Apr 13;16(4):e1008629. doi: 10.1371/journal.pgen.1008629 (PMC7200007; doi:10.1371/journal.pgen.1008629)
Supplement: S8 Table — (DOCX) [file pgen.1008629.s008.docx]

Supplementary Table 8. Definition of outcomes in phenome wide association study in UK Biobank.

| **Outcome** | **Definition (UK Biobank unless otherwise specified)** |
| --- | --- |
| Coronary artery disease | (1) Myocardial infarction (MI), coronary artery bypass grafting, or coronary artery angioplasty documented in medical history at time of enrollment by a trained nurse or  (2) Hospitalization for ICD-10 code for acute myocardial infarction (I21.0, I21.1, I21.2, I21.4, I21.9) or  (3) Hospitalization for OPCS-4 coded procedure: coronary artery bypass grafting (K40.1-40.4, K41.1-41.4, K45.1-45.5) or  (4) Hospitalization for OPCS-4 coded procedure: coronary angioplasty ± stenting (K49.1-49.2, K49.8-49.9, K50.2, K75.1-75.4, K75.8-75.9) |
| Atrial fibrillation/flutter | History of atrial fibrillation or flutter during verbal interview with trained nurse or hospitalization for or death due to ICD code I48 |
| Heart failure | History of heart failure during verbal interview with trained nurse or hospitalization for or death due to ICD code I11.0, I13.0, I13.2, I125.5, I42, I50 |
| Stroke | History of stroke, adjudicated by UK Biobank centrally as report of stroke during verbal interview with trained nurse or hospitalization for or death due to ICD code I60-64 *(http://biobank.ctsu.ox.ac.uk/crystal/refer.cgi?id=462)* |
| Peripheral vascular disease | History of peripheral vascular disease or intermittent claudication during verbal interview with trained nurse or hospitalization for or death due to ICD code I70, I73.8 or I73.9 |
| Venous thromboembolism | History of venous thromboembolism, deep vein thrombosis or pulmonary embolism during verbal interview with trained nurse or hospitalization for death due to I26, I80.1, I80.2, I81, or I82.0 |
| Aortic stenosis | History of aortic stenosis during verbal interview with trained nurse or hospitalization for ICD code I06.0, I06.2 I35.0 or I35.2 |
| Inflammatory bowel disease | History of inflammatory bowel disease, Crohn’s disease or ulcerative colitis during verbal interview with trained nurse or hospitalization for or death due to ICD code K50 or K51 |
| Gastric reflux | History of gastric reflux during verbal interview with trained nurse or hospitalization for or death due to ICD code K21 |
| Gallstone | History of gallstones during verbal interview with trained nurse or hospitalization for or death due to ICD code K56.3 or K80 |
| Type 2 Diabetes | History of diabetes unspecified, type 2 diabetes during verbal interview with trained nurse or hospitalization for or death due to ICD code E11 |
| Hyperthyroidism | History of hyperthyroidism during verbal interview with trained nurse or hospitalization for or death due to ICD code E05 |
| Hypothyroidism | History of hypothyroidism during verbal interview with trained nurse or hospitalization for or death due to ICD code E03 |
| Gout | History of gout during verbal interview with trained nurse or hospitalization for or death due to ICD code M10 |
| Enlarged prostate | History of enlarged prostate during verbal interview with trained nurse or hospitalization for or death due to ICD code N40 |
| Uterine fibroids | History of uterine fibroids during verbal interview with trained nurse or hospitalization for or death due to ICD code D25 |
| Migraine | History of migraine during verbal interview with trained nurse or hospitalization for or death due to ICD code G43 |
| Depression | History of depression during verbal interview with trained nurse or hospitalization for or death due to ICD code F32 |
| Anxiety | History of anxiety/panic attacks during verbal interview with trained nurse or hospitalization for or death due to ICD code F41 |
| Osteoporosis | History of osteoporosis during verbal interview with trained nurse or hospitalization for or death due to ICD code M80 or M81 |
| Osteoarthritis | History of osteoarthritis during verbal interview with trained nurse or hospitalization for or death due to ICD code M15-19 |
| Sciatica | History of sciatica during verbal interview with trained nurse or hospitalization for or death due to ICD code M54.3 |
| Prolapsed disc | History of prolapsed disc/slipped disc during verbal interview with trained nurse or hospitalization for or death due to ICD code M50.2 or M51.2 |
| Asthma | History of asthma during verbal interview with trained nurse or hospitalization for or death due to ICD code J45 or J46 |
| COPD/Emphysema | History of chronic obstructive airways disease, emphysema/chronic bronchitis or emphysema during verbal interview with trained nurse or hospitalization for or death due to ICD code J41-44 |
| Pneumonia | History of pneumonia during verbal interview with trained nurse or hospitalization for or death due to ICD code J12-18 |
| Hayfever | History of hayfever during verbal interview with trained nurse or hospitalization for or death due to ICD code J30 |
| Lung cancer | History of lung cancer during verbal interview with trained nurse or hospitalization for or death due to ICD code C34 |
| Colorectal cancer | History of large bowel cancer/colorectal cancer, colon cancer/sigmoid cancer or rectal cancer during verbal interview with trained nurse or hospitalization for or death due to ICD code C18 |
| Skin cancer | History of skin cancer, malignant melanoma, non-melanoma skin cnacer, basal cell carcinoma or squamous cell carcinoma during verbal interview with trained nurse or hospitalization for or death due to ICD code C43-44 |
| Prostate cancer | History of prostate cancer during verbal interview with trained nurse or hospitalization for or death due to ICD code C61 |
| Cervical cancer | History of cervical cancer or cin cells at the cervix during verbal interview with trained nurse or hospitalization for or death due to ICD code C53 |

Abbreviations: COPD, chronic obstructive pulmonary disease; ICD, international classification of disease
