# Supplementary material for: A missense variant in Mitochondrial Amidoxime Reducing Component 1 gene and protection against liver disease
Source: PLoS Genet. 2020 Apr 13;16(4):e1008629. doi: 10.1371/journal.pgen.1008629 (PMC7200007; doi:10.1371/journal.pgen.1008629)
Supplement: S2 Fig — (PDF) [file pgen.1008629.s010.pdf]

Type of Cirrhosis	Cases	Controls	OR	95% CI	p–value
**PNPLA3 I48M**					
All–cause Cirrhosis	1740	403829	1.39	[1.29; 1.50]	4.9e–18
Alcoholic Cirrhosis	550	405019	1.58	[1.39; 1.80]	4.6e–12
Non–Alcoholic Cirrhosis	991	404379	1.36	[1.23; 1.50]	1.6e–09
**TM6SF2 E167K**					
All–cause Cirrhosis	1740	403829	1.48	[1.33; 1.65]	1.7e–12
Alcoholic Cirrhosis	550	405019	1.47	[1.21; 1.79]	9.2e–05
Non–Alcoholic Cirrhosis	991	404379	1.52	[1.31; 1.75]	1.1e–08
**MBOAT7 rs641738**					
All–cause Cirrhosis	1740	403829	1.17	[1.06; 1.30]	0.0031
Alcoholic Cirrhosis	550	405019	1.32	[1.07; 1.62]	0.0087
Non–Alcoholic Cirrhosis	991	404379	1.14	[1.00; 1.31]	0.053
**HSD17B13 rs72613567**					
All–cause Cirrhosis	1740	403829	0.82	[0.76; 0.89]	8.6e–07
Alcoholic Cirrhosis	550	405019	0.85	[0.74; 0.98]	0.023
Non–Alcoholic Cirrhosis	991	404379	0.80	[0.72; 0.89]	3.1e–05
**HFE C282Y**					
All–cause Cirrhosis	1740	403829	1.37	[1.22; 1.53]	5e–08
Alcoholic Cirrhosis	550	405019	1.36	[1.12; 1.65]	0.0022
Non–Alcoholic Cirrhosis	991	404379	1.36	[1.17; 1.58]	8.2e–05
**SERPINA1 E366K**					
All–cause Cirrhosis	1740	403829	1.33	[1.07; 1.65]	0.0097
Alcoholic Cirrhosis	550	405019	1.18	[0.79; 1.78]	0.41
Non–Alcoholic Cirrhosis	991	404379	1.42	[1.07; 1.87]	0.015

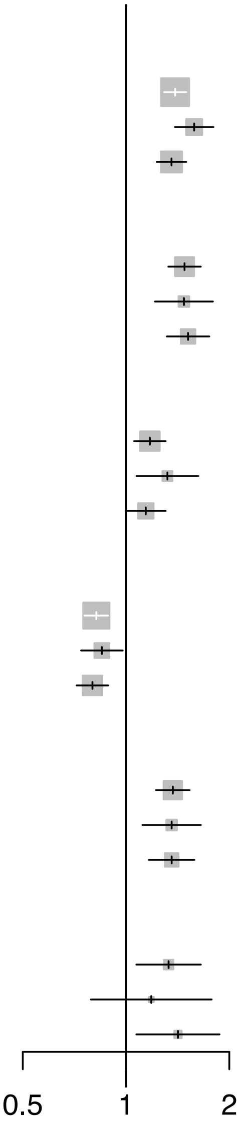

Supplementary Figure 2. Association of known alcoholic and non-alcoholic cirrhosis variants with all-cause cirrhosis in UK Biobank.
