# Supplementary figures and images for: A missense variant in Mitochondrial Amidoxime Reducing Component 1 gene and protection against liver disease
Source: PLoS Genet. 2020 Apr 13;16(4):e1008629. doi: 10.1371/journal.pgen.1008629 (PMC7200007; doi:10.1371/journal.pgen.1008629)

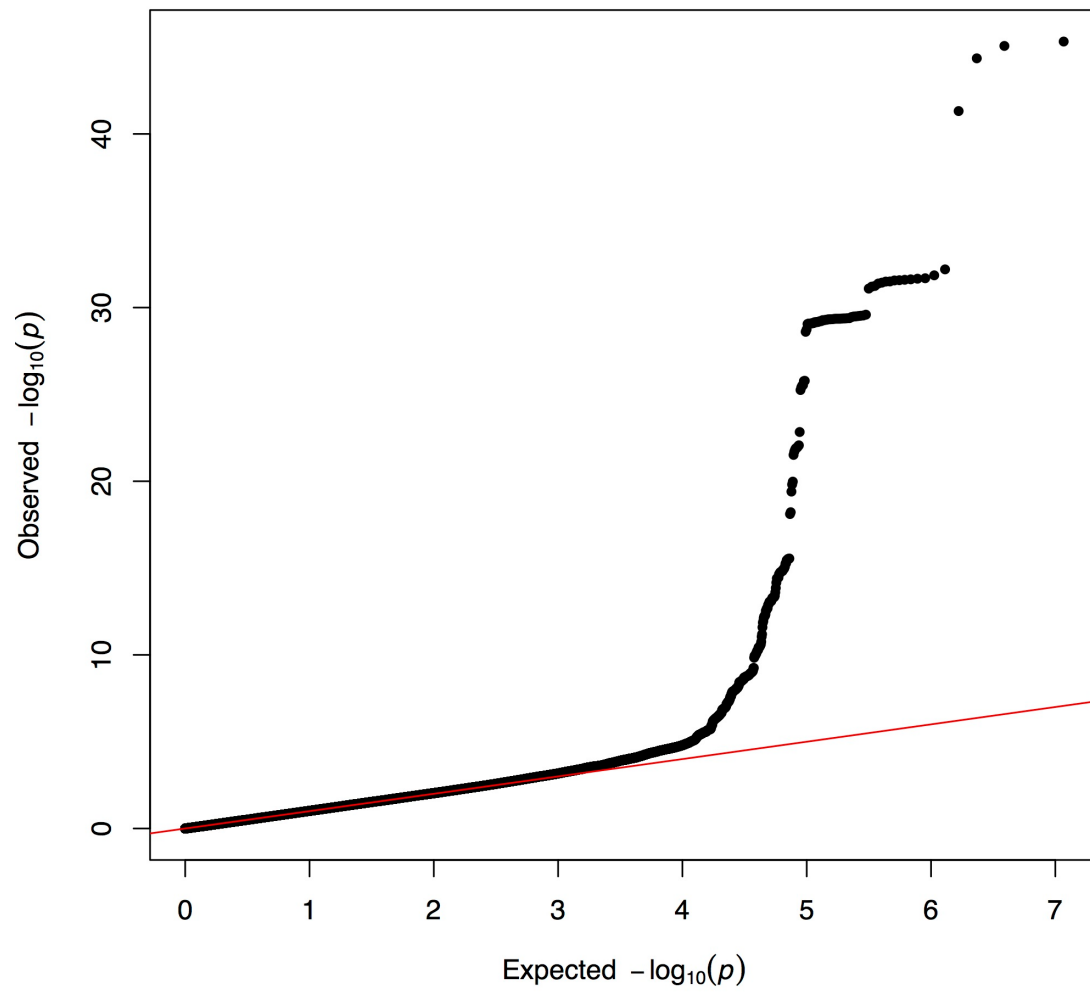

Supplementary Figure 3. QQ plot for genome wide analysis of cirrhosis. Lambda = 1.02

Supplement: S3 Fig — (PDF) [file pgen.1008629.s011.pdf]
