# Supplementary material for: A missense variant in Mitochondrial Amidoxime Reducing Component 1 gene and protection against liver disease
Source: PLoS Genet. 2020 Apr 13;16(4):e1008629. doi: 10.1371/journal.pgen.1008629 (PMC7200007; doi:10.1371/journal.pgen.1008629)
Supplement: S4 Fig — (PDF) [file pgen.1008629.s012.pdf]

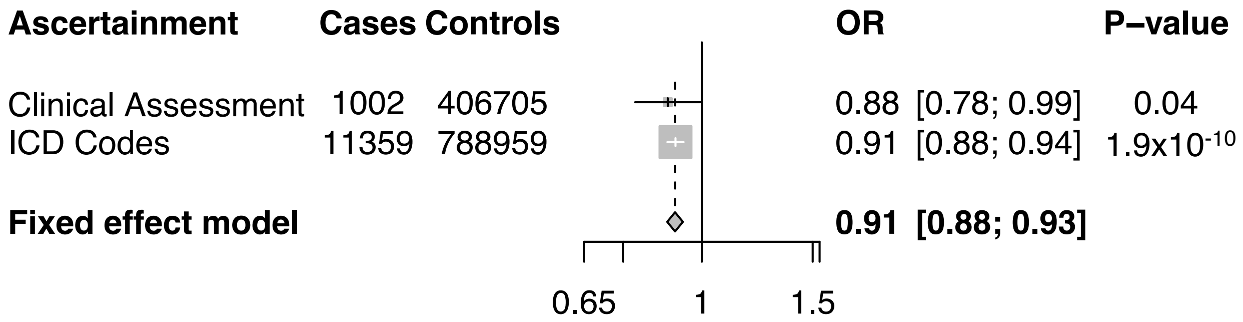

Supplementary Figure 4. Risk of cirrhosis associated with *MARC1* A165T among cohorts by method of cirrhosis ascertainment.
