# Supplementary material for: A missense variant in Mitochondrial Amidoxime Reducing Component 1 gene and protection against liver disease
Source: PLoS Genet. 2020 Apr 13;16(4):e1008629. doi: 10.1371/journal.pgen.1008629 (PMC7200007; doi:10.1371/journal.pgen.1008629)
Supplement: S5 Fig — (PDF) [file pgen.1008629.s013.pdf]

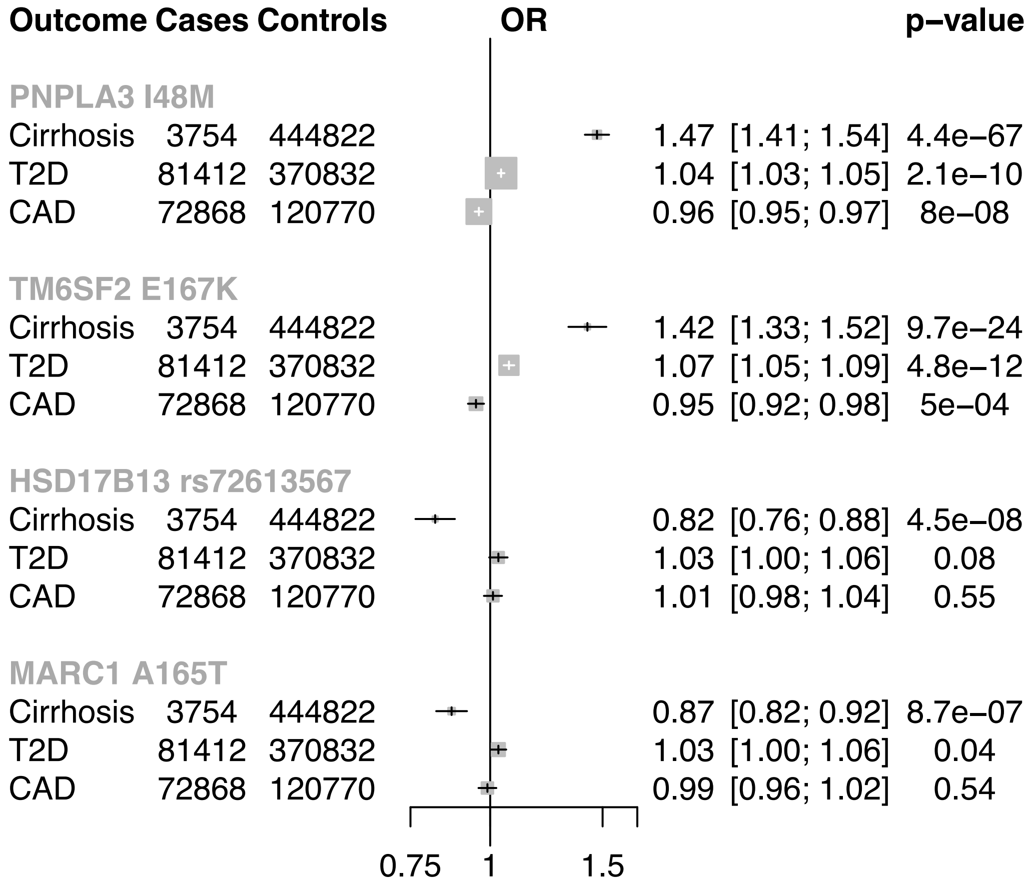

Supplementary Figure 5. Association of cirrhosis variants with type 2 diabetes, coronary artery disease and cirrhosis.
