# Supplementary material for: A missense variant in Mitochondrial Amidoxime Reducing Component 1 gene and protection against liver disease
Source: PLoS Genet. 2020 Apr 13;16(4):e1008629. doi: 10.1371/journal.pgen.1008629 (PMC7200007; doi:10.1371/journal.pgen.1008629)
Supplement: S6 Fig — (PDF) [file pgen.1008629.s014.pdf]

Outcome	Cases	Controls	OR		p–value
**Cardiovascular**					
Coronary Artery Disease	14811	390758	1.01	[0.98; 1.04]	0.49
Stroke	9227	396342	1.00	[0.97; 1.04]	0.85
Heart Failure	6039	399530	1.02	[0.98; 1.06]	0.31
Atrial Fibrillation	14623	390946	1.01	[0.98; 1.04]	0.47
Aortic Stenosis	1850	403719	0.96	[0.89; 1.03]	0.26
Peripheral vascular disease	4720	400849	1.03	[0.98; 1.08]	0.21
Venous thromboembolism	13281	392288	1.00	[0.97; 1.03]	0.87
**Gastrointestinal disease**					
Inflammatory bowel disease	5400	400169	0.96	[0.92; 1.01]	0.092
Gastric reflux	35568	370001	1.00	[0.99; 1.02]	0.82
Gallstones	17780	387789	0.96	[0.94; 0.98]	0.00064
**Endocrine**					
Type 2 Diabetes	20458	385111	1.00	[0.98; 1.02]	0.85
Hyperthyroidium	4477	401092	1.01	[0.97; 1.06]	0.65
Hypothyroidism	23591	381978	0.99	[0.97; 1.01]	0.55
Gout	7382	398187	1.06	[1.02; 1.10]	0.0013
**Urological**					
Enlarged Prostate	14323	391246	0.99	[0.96; 1.01]	0.3
Uterine Fibroids	17128	388441	0.98	[0.96; 1.01]	0.17
**Neurological/psychiatric**					
Migraine	14293	391276	1.00	[0.97; 1.02]	0.84
Depression	30835	374734	1.02	[1.00; 1.04]	0.07
Anxiety	6733	398836	1.02	[0.98; 1.06]	0.3
**Muscoskeletal**					
Osteoporosis	10863	394706	0.98	[0.95; 1.01]	0.29
Osteoarthritis	62643	342926	1.02	[1.00; 1.03]	0.01
Sciatica	6222	399347	0.98	[0.95; 1.02]	0.44
Prolapsed disc	9463	396106	1.01	[0.98; 1.05]	0.39
**Respiratory**					
Asthma	47179	358390	1.01	[0.99; 1.02]	0.49
COPD/Emphysema	13771	391798	0.99	[0.97; 1.02]	0.6
Pneumonia	14869	390700	0.99	[0.96; 1.01]	0.34
Hayfever	25489	380080	1.00	[0.98; 1.02]	0.66
**Cancer**					
Lung Cancer	2281	403288	1.02	[0.96; 1.09]	0.57
Colorectal Cancer	3969	401600	0.99	[0.95; 1.04]	0.78
Skin Cancer	16904	388665	0.99	[0.97; 1.02]	0.65
Prostate Cancer	6377	399192	1.04	[1.00; 1.08]	0.046
Cervical Cancer	1707	403862	1.05	[0.98; 1.13]	0.17

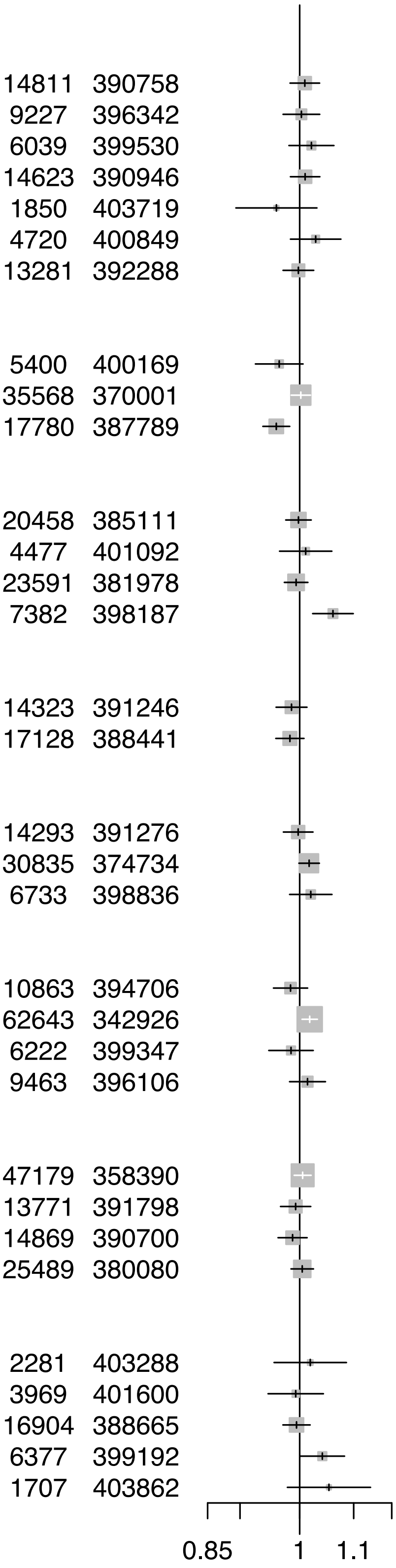

Supplementary Figure 6. Association of MARC A165T with other diseases in a phenome wide association study.
