# Supplementary material for: Correction: A missense variant in Mitochondrial Amidoxime Reducing Component 1 gene and protection against liver disease
Source: PLoS Genet. 2021 Apr 6;17(4):e1009503. doi: 10.1371/journal.pgen.1009503 (PMC8023447; doi:10.1371/journal.pgen.1009503)
Supplement: S1 Fig — (PDF) [file pgen.1009503.s001.pdf]

Type of Cirrhosis	Source	Cases	Controls	OR	95% CI	p−value
**PNPLA3 I148M**						
Alcoholic Cirrhosis	Buch et al. [1]	1860	2348	2.39	[2.05; 2.78]	1.5e−48
Non−Alcoholic Cirrhosis	Speliotes et al. [2]	533	1405	3.37	[2.86; 3.98]	3.6e−46
Hepatitis C Cirrhosis	Valenti et al. [3]	215	604	1.47	[1.15; 1.87]	0.002
**TM6SF2 E167K**						
Alcoholic Cirrhosis	Buch et al. [1]	1860	2348	1.84	[1.42; 2.39]	7.9e−10
Non−Alcoholic Cirrhosis	Liu et al. [4]	437	637	1.88	[1.41; 2.50]	1.6e−05
Hepatitis C Cirrhosis	Liu et al. [5]	.	.	2.05	[1.39; 3.02]	3e−04
**HSD17B13 rs72613567:TA**						
Alcoholic Cirrhosis	Abul−Husn et al. [6]	124	29928	0.56	[0.41; 0.77]	0.00034
Non−Alcoholic Cirrhosis	Abul−Husn et al. [6]	374	29928	0.74	[0.62; 0.88]	0.00048
Hepatitis C Cirrhosis	About et al. [7]	40	48	0.38	[0.16; 0.91]	0.03

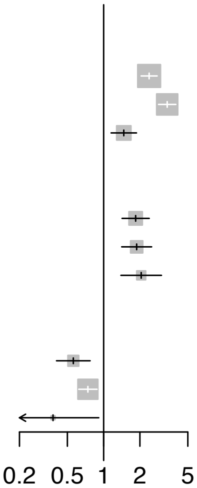

Supplementary Figure 1. Risk of alcoholic, non-alcoholic and hepatitis C cirrhosis associated with PNPLA3 I148M, TM6SF2 E167K and HSD17B13.[1-7] Valenti et al. reports results from a recessive model.

References

1. Buch S, Stickel F, Trépo E, et al. A genome-wide association study confirms PNPLA3 and identifies TM6SF2 and MBOAT7 as risk loci for alcohol-related cirrhosis. *Nat Genet*. 2015;47(12):1443-1448. doi:10.1038/ng.3417.
2. Speliotes EK, Butler JL, Palmer CD, et al. PNPLA3 variants specifically confer increased risk for histologic nonalcoholic fatty liver disease but not metabolic disease. *Hepatology*. 2010;52(3):904-912. doi:10.1002/hep.23768.
3. Valenti L, Rumi M, Galmozzi E, et al. Patatin-like phospholipase domain-containing 3 I148M polymorphism, steatosis, and liver damage in chronic hepatitis C. *Hepatology*. 2011;53(3):791-799. doi:10.1002/hep.24123.
4. Liu Y-L, Reeves HL, Burt AD, et al. TM6SF2 rs58542926 influences hepatic fibrosis progression in patients with non-alcoholic fatty liver disease. *Nat Commun*. 2014;5(1):4309. doi:10.1038/ncomms5309.
5. Liu Z, Que S, Zhou L, et al. The effect of the TM6SF2 E167K variant on liver steatosis and fibrosis in patients with chronic hepatitis C: a meta-analysis. *Sci Rep*. 2017;7(1):9273. doi:10.1038/s41598-017-09548-9.
6. Abul-Husn NS, Cheng X, Li AH, et al. A Protein-Truncating HSD17B13 Variant and Protection from Chronic Liver Disease. *N Engl J Med*. 2018;378(12):1096-1106. doi:10.1056/NEJMoa1712191.
7. About F, Abel L, Cobat A. HCV-Associated Liver Fibrosis and HSD17B13. *N Engl J Med*. 2018;379(19):1875-1876. doi:10.1056/NEJMc1804638.
